# Dyspepsia

**Published:** 1874-06

**Authors:** 


### DYSPEPSIA.

THE only cure for this prevalent malady is the proper regulation of the food; first let it be regularly taken; let it be in moderate amounts; let it be well cooked, plain and nutritious, and easily digested; this, with a moderate degree of gentle exercise in the open air two or three hours a day, will cure any ordinary case. Dyspeptics always get well and get fat when they are sent to the penitentiary for a year or two, because their food is plain, is taken regularly, and the exercise is moderate, steady and healthful. If a man "can't" diet himself thus, then let him suffer, or go to jail.

